# Impact of Etiology, Thrombectomy Techniques, and Lesion Localization on Endovascular Treatment Success in Acute Basilar Artery Occlusion: A Single‐Center Retrospective Study

**DOI:** 10.1002/brb3.70612

**Published:** 2025-06-06

**Authors:** Halil Alper Eryilmaz, Sena Boncuk Ulaş, Bilgehan Atılgan Acar

**Affiliations:** ^1^ Department of Neurology Sakarya Training and Research Hospital Sakarya Turkey; ^2^ Independent Researcher (MD, Neurologist) Dunkerque France; ^3^ Department of Neurology Sakarya University Faculty of Medicine Sakarya Turkey

**Keywords:** basilar artery occlusion, clinical outcomes, endovascular treatment, recanalization, stroke, thrombectomy

## Abstract

**Background:**

Basilar artery occlusion (BAO) is a rare but severe form of large vessel occlusion associated with high rates of mortality and morbidity. Endovascular treatment (EVT) has become the primary therapeutic option; however, its efficacy and procedural risks in BAO remain controversial. This study aims to evaluate the clinical and procedural outcomes of EVT in BAO and to compare these results across different etiological subgroups.

**Methods:**

We retrospectively analyzed 23 patients who underwent EVT for BAO between December 2019 and December 2024. Demographic data, procedural times, recanalization success rates, complications, and functional outcomes assessed by the Modified Rankin Scale (mRS) at three months were evaluated. The patients were categorized into three groups based on the etiology: large artery atherosclerosis, cardioembolism, and undetermined causes.

**Results:**

Successful recanalization (mTICI 2c‐3) was achieved in 100% of cases. The mean symptom‐to‐recanalization time was the longest in the large artery atherosclerosis group (345 ± 165.38 min). At the three‐month follow‐up, 47.8% of patients had a good functional outcome (mRS 0–2), and 34.1% achieved an excellent outcome (mRS 0–1). The cardioembolism group had lower mean mRS scores compared to other groups. Intracranial hemorrhage occurred in 13% of cases, with 8.7% classified as symptomatic.

**Conclusion:**

The mean 3‐month mRS scores were lower in the cardioembolic etiology group. Although the combined technique group had longer recanalization times, they exhibited lower mean 3rd month mRS scores. As the number of randomized controlled trials increases, it is expected that EVT in BAO will be recommended with higher levels of evidence in the guidelines.

## Graphical abstract

In acute basilar artery occlusion, endovascular treatment achieved 100% recanalization, with lower 3‐month mRS observed in the cardioembolic group and those treated with combined techniques despite longer procedure times. Larger, randomized controlled studies are needed to confirm and clarify the strength of our findings.

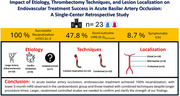

## 1 Introduction

Acute basilar artery occlusion (BAO) is a rare but clinically significant condition that can lead to ischemia in the brainstem, occipital lobes, and thalamus, resulting in severe neurological consequences (Yu and Higashida 2022; Alemseged et al. 2023; Kocayigit et al. 2022). Without prompt reperfusion, the rate of mortality and/or disability can reach approximately 90% (Yu and Higashida 2022). The efficacy of endovascular treatment (EVT) in BAO remains uncertain and continues to be a topic of debate (Alemseged et al. 2023; Kocayigit et al. 2022; Akdağ et al. 2024; Alemseged et al. 2023). According to the 2019 guidelines of the American Heart Association and the American Stroke Association, EVT within the first six hours in patients with BAO has not been conclusively proven to improve functional outcomes and is recommended with a Class IIb evidence level C (Powers et al. 2019).

However, two randomized controlled trials (BEST and BASICS) conducted in 2020 and 2021 demonstrated that EVT did not provide a significant functional advantage compared to medical management (Liu et al. 2020; Langezaal et al. 2021). Following these findings, two large randomized controlled trials published in 2022 (ATTENTION and BAOCHE) indicated that EVT performed within the first 24 h of BAO onset was superior to medical treatment in terms of functional outcomes (Tao et al. 2022; Jovin et al. 2022). Nevertheless, both studies highlighted that EVT was associated with procedural complications and a higher incidence of intracranial hemorrhage. Furthermore, a meta‐analysis published in January 2025, which evaluated the efficacy of EVT in BAO by combining data from these four major trials, concluded that EVT significantly reduces mortality and morbidity but is also associated with an increased risk of symptomatic intracranial hemorrhage (Nogueira et al. 2025).

In this study, we aimed to evaluate the efficacy of EVT in BAO by analyzing the data from our center's experience and comparing our findings with the existing literature.

## 2 Materials and Methods

Patient data from the Stroke Center of the Neurology Clinic at Sakarya University Training and Research Hospital were retrospectively reviewed for patients who underwent EVT between December 2019 and December 2024. Prior to the initiation of the study, ethical approval was obtained from our university's ethics committee (approval number: E‐43012747‐050.04‐438109‐06). Patients who underwent EVT due to posterior circulation large artery occlusion during the specified period were included in the study. Patients were selected retrospectively from a prospectively maintained institutional stroke registry. Only those with complete clinical, radiological, and procedural data were included in the final analysis. However, since there were no missing data in our dataset, no patients needed to be excluded from the study. A total of 23 cases were included in the study.

The demographic data (age, gender), comorbidities [hypertension (HT), diabetes mellitus (DM), atrial fibrillation (AF), coronary artery disease (CAD), coronary artery bypass grafting (CABG), history of previous stroke, smoking, substance abuse, alcoholism], and use of antiplatelet and anticoagulant therapies were collected from patient records. Stroke etiologies were classified into three groups based on the Trial of Org 10172 in Acute Stroke Treatment (TOAST) criteria (Adams et al. 1993): large‐artery atherosclerosis, cardioembolic, and undetermined. Patients with multiple etiologies or those who did not undergo advanced cardiac evaluation due to death were categorized as having an undetermined etiology.

Clinical data collected included the National Institutes of Health Stroke Scale (NIHSS) (Rajashekar et al. 2022) scores at admission and on the first day, blood pressure and glucose levels measured in the emergency department, and lipid parameters [LDL (mg/dL), HDL (mg/dL), triglycerides (mg/dL)] within the first 48 h. Procedural time metrics, including door‐to‐puncture time, puncture‐to‐recanalization time, and symptom‐to‐recanalization time, were also recorded. Successful recanalization status, first‐pass recanalization status, thrombectomy techniques used, the application of anesthesia, and hemorrhagic transformation within the first 24 h (classified according to the Heidelberg bleeding classification) (von Kummer et al. 2015) were documented. Functional outcomes were assessed using the Modified Rankin Scale (mRS) at 3 months. Successful recanalization was defined according to the Modified Thrombolysis in Cerebral Infarction (mTICI) Scale (Elawady et al. 2024).

Descriptive statistical analyses included mean, standard deviation, median, minimum, maximum, frequency, and percentage values. For the analysis of quantitative independent variables, the independent samples *t*‐test, Kruskal–Wallis test, and Mann‐Whitney *U* test were applied. For the analysis of qualitative independent variables, the chi‐square test was used; if chi‐square test conditions were not met, Fisher's exact test was applied. Statistical analyses were performed using the SPSS 27.0 software package.

## 3 Results

Among the 23 cases who underwent EVT for acute BAO, 73.9% were male. The age range of the cases was between 29 and 83 years, with a mean age of 60.86±12.87 years. The NIHSS scores at hospital admission ranged from 2 to 38, with a mean of 20 ± 8.09, and 87% of the cases had NIHSS scores ≥ 10. Regarding medical history, the most common risk factor was HT at 60.9% (14 cases). In addition, 21.7% (5 cases) had type 2 DM, 17.4% (4 cases) had CAD, 13% (3 cases) had a history of CABG, and 13% (3 cases) had a previous stroke. AF was observed in 26.1% (6 cases). Active smoking was reported in 34.8% (8 cases), and chronic alcoholism in 13% (3 cases). Antiplatelet therapy was used by 30.4% (7 cases). None of the cases had a history of cancer. At emergency department admission, the mean systolic and diastolic blood pressures were 165.59 ± 26.69 mmHg and 92.56 ± 16.25 mmHg, respectively. The mean blood glucose level was 163.21 ± 61.27 mg/dL. The mean LDL, HDL, and triglyceride levels were 127.13 ± 35.93 mg/dL, 41.60 ± 10.39 mg/dL, and 128.78 ± 112.75 mg/dL, respectively. According to the TOAST classification, 43.5% (10 cases) were categorized as large artery atherosclerosis, 26.1% (6 cases) as cardioembolism, and 30.4% (7 cases) as undetermined etiology (Table 1).

**TABLE 1:** Demographic characteristics, comorbidities, and laboratory parametres of cases with acute basilar artery occlusion.

		Min–Max	Mean±Sd	*n*	%
Sex	Female			6	26.1%
	Male			17	73.9%
Age		29–83	60.86 ± 12.87		
Admission NIHSS		2–38	20 ± 8.09		
NIHSS ≥ 10				20	87%
Comorbidities					
Atrial fibrillation (+)				6	26.1%
Hypertension (+)				14	60.9%
Smoking (+)				8	34.8%
Substance abuse (+)				1	4.3%
Alcoholism (+)				3	13%
Obesity (+)				2	8.7%
Type 2 DM (+)				5	21.7%
CABG (+)				3	13%
CAD (+)				4	17.4%
MI in the last 3 month (+)				0	0%
Previous stroke (+)				3	13%
Malignancy (+)				0	0%
Antiagregant medication (+)				7	30.4%
Admission systolic BP (mmHg)		100–210	165.59 ± 26.69		
Admission diastolic BP (mmHg)		70–140	92.56 ± 16.25		
Admission glucose (mg/dL)		85–307	163.21 ± 61.27		
LDL (mg/dL)		70–215	127.13 ± 35.93		
HDL (mg/dL)		23–59	4160 ± 10.39		
Triglyceride (mg/dL)		42–499	128.78 ± 112.75		
Etiology		Large artery aterosclerosis		10	43.5%
		Cardioembolism		6	26.1%
		Undetermined		7	30.4%

Abbreviations: BP, blood pressure; CABG, coronary artery bypass grafting; CAD, coronary artery disease; DM, diabetes mellitus; HDL, high‐density lipoprotein; LDL, low‐density lipoprotein; Max, maximum; MI, myocardial infarction; Min, minimum; NIHSS, National Institute of Health Stroke Scale; Sd, standard deviation.

Regarding the occlusion site, 21.7% (5 cases) had proximal BAO, while 78.3% (18 cases) had distal BAO. Mid‐basilar occlusion was not observed. In addition, 17.4% (4 cases) had concomitant vertebral artery occlusion. The most common aortic arch type was Type 2 at 69.6%, and the bovine arch was present in 13% of the cases. The mean door‐to‐puncture, puncture‐to‐microcatheter, and puncture‐to‐recanalization times were 92.56 ± 54.03 min, 43.21 ± 19.28 min, and 64.82 ± 28.04 min, respectively. Symptom‐to‐recanalization times ranged from 125 to 636 min, with a mean of 295.78 ± 128.70 min. Post‐procedurally, mTICI 2c recanalization was achieved in 1 case, and mTICI 3 recanalization was achieved in 22 cases, resulting in a 100% successful recanalization rate. First‐pass successful recanalization was achieved in 60.9% (14 cases) (Table 2).

**TABLE 2:** DSA‐related parameters, procedural complications, and mRS values of cases with acute basilar artery occlusion.

		Min–Max	Mean ± Sd	*n*	%
Localization of occluded artery	Proximal basilar artery			5	21.7%
Distal basilar artery				18	78.3%
Bovine arch (+)			3	13%	
Vertebral artery occlusion			4	17.4%	
Time metrics (minutes)					
Door‐to‐puncture time		43–256	92.56 ± 54.03		
Puncture‐to‐microcatather time		10–91	43.21 ± 19.28		
Puncture‐to‐recanalization Time		13–146	64.82 ± 28.04		
Symptom‐to‐recanalization Time		125–636	295.78 ± 128.70		
Final successful recanalization (mTICI 2c‐3)				23	100%
First‐pass successful recanalization (mTICI 2c‐3)				14	60.9%
EVT techniques		Combined techniques (Aspiration + Stent retriever)		10	43.5%
		Contact aspiration		13	56.5%
Total intracranial pass number		1		14	60.9%
		2		6	26.1%
		3		2	8.7%
		4		1	4.3%
Anesthesia type		General anesthesia with intubation		8	34.8%
		Conscious sedation		2	8.7%
		Local anesthesia		13	56.5%
Intracerebral hemorrhage in first 24 h				3	13%
3rd month mRS		0–6	3.04 ± 2.4		
3rd month 0–2 (+)				11	47.8%
3rd month 0–1 (+)				9	39.1%
3rd month 0–3 (+)				12	52.2%
3rd month 6 (+)				7	30.4%

Abbreviations: EVT, endovascular treatment; h, hour; Max, maximum; Min, minimum; mRS, Modified Rankin Scale; mTICI, Modified Thrombolysis in Cerebral Infarction; Sd, standard deviation.

**FIGURE 1:**
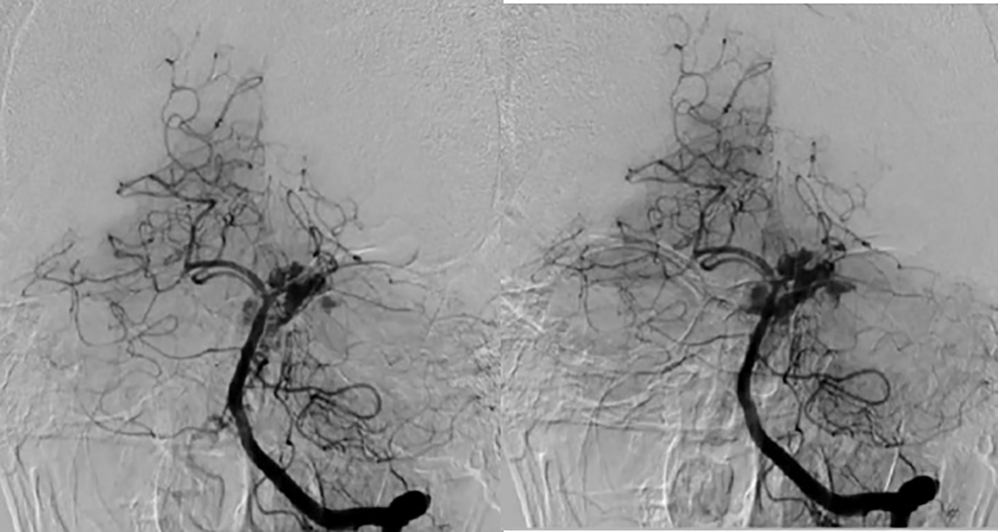
DSA imaging of intracranial hemorrhage due to left PCA rüptüre after Stent retriever removal.

In terms of EVT techniques, 56.5% (13 cases) underwent contact aspiration, while 43.5% (10 cases) underwent a combined technique (aspiration + stent retriever) for recanalization (Table 2). The number of intracranial passes ranged from 1 to 4, with 60.9% of cases requiring only 1 intracranial pass. General anesthesia was applied in 34.8% (8 cases), and conscious sedation in 8.7% (2 cases). Regarding procedural complications, distal embolism occurred in only 1 case (4.3%), and no cases of vasospasm were observed. Vertebral artery rupture and hemorrhage occurred in 1 case during balloon angioplasty for inadequate flow restoration in the proximal basilar artery. Another case experienced intracranial hemorrhage due to left posterior cerebral artery (PCA) rupture after stent retriever removal (Figure 1). In total, intracranial hemorrhage was detected in 3 cases (13%), comprising 1 case of Type 2 hemorrhagic infarction, 1 case of Type 1 parenchymal hematoma, and 1 case of subarachnoid hemorrhage, according to the Heidelberg Bleeding Classification. Symptomatic intracranial hemorrhage occurred in 2 cases (8.7%).

Based on the mRS scores at 3 months, 47.8% (11 cases) achieved a good clinical outcome (mRS 0–2), while 39.1% (9 cases) had an mRS score of 0–1. The mean mRS score at 3 months was 3.04 ± 2.4 (Table 2). In cases with an mRS score of 0–1, the mean puncture‐to‐recanalization and symptom‐to‐recanalization times were 67.0 ± 23.48 and 296.0 ± 137.74 min, respectively, while these times were 69.70 ± 22.72 and 301.70 ± 134.14 min in cases with an mRS score of 0–2.

**FIGURE 2:**
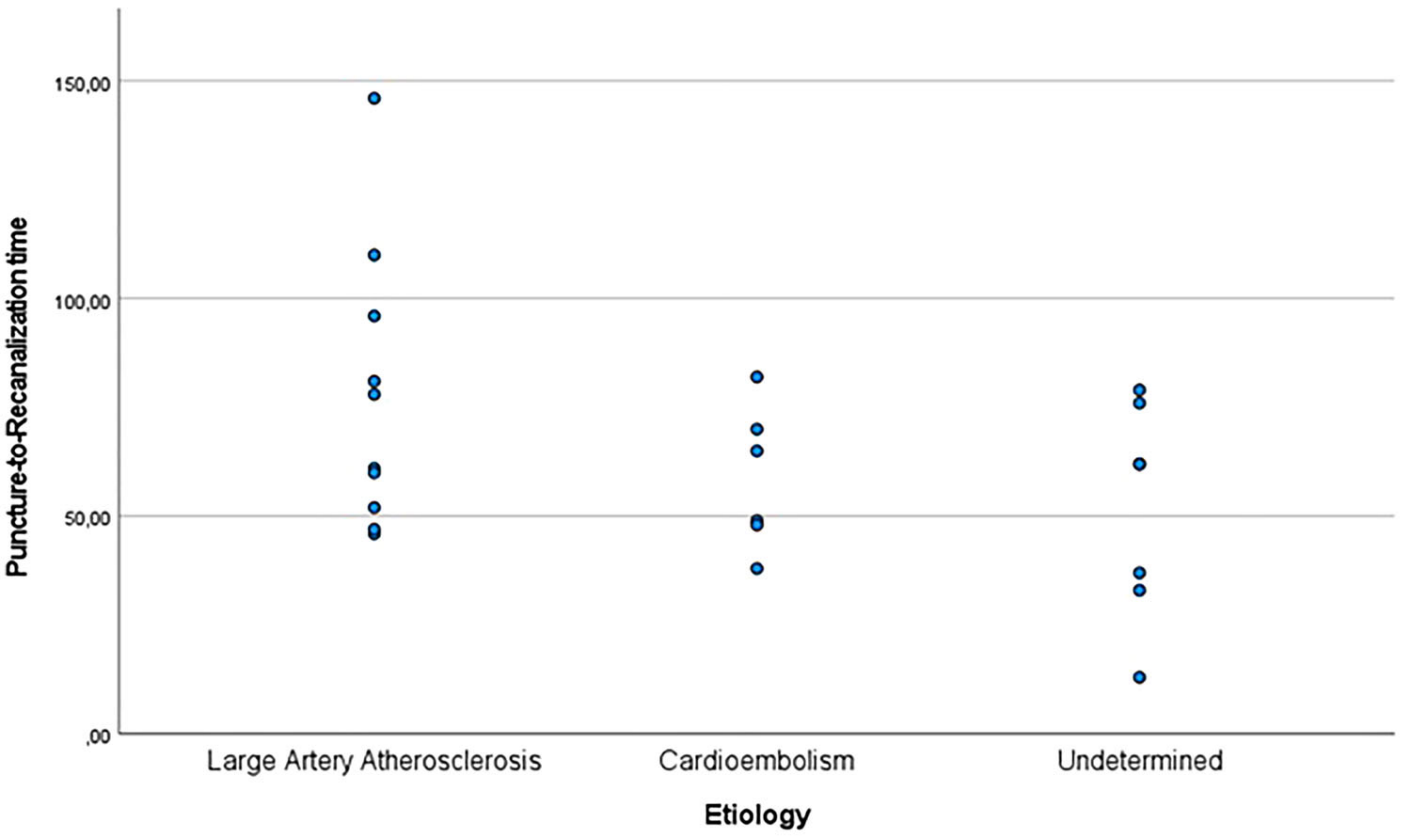
Puncture‐to‐recanalization time according to the etiology of the cases.

When cases were categorized by etiology, 34.8% of the large artery atherosclerosis group, 13% of the cardioembolism group, and 30.4% of the undetermined etiology group had distal BAO. Proximal BAO was observed in 8.7% of the large artery atherosclerosis group and 13% of the cardioembolism group. Although the puncture‐to‐recanalization and symptom‐to‐recanalization times were longer in the large artery atherosclerosis group compared to the other groups, no statistically significant differences were found (Figure 2). When evaluating thrombectomy techniques by etiology, contact aspiration and combined technique were each used in 21.7% of the cases in the large artery atherosclerosis group. In the cardioembolism group, the rates were 17.4% and 8.7%, respectively; and in the undetermined etiology group, 17.4% and 13%. Regarding recanalization outcomes, all patients achieved successful recanalization, with one case (4.3%) in the cardioembolism group having mTICI 2c and the remainder having mTICI 3 (Table 3).

**TABLE 3:** DSA parameters and clinical outcomes according to the etiology of cases with acute basilar artery occlusion.

	Large artery aterosclerosis (*n* = 10)	Cardioembolism (*n* = 6)	Undetermined (*n* = 7)	*p*
Localization of occluded artery				
Distal basilar artery occlusion	8 (34.8%)	3 (13%)	7 (30.4%)	0.056
Proximal basilar artery occlusion	2 (8.7%)	3 (13%)	0 (0%)	
Time metrics (minutes)				
Mean puncture‐to‐recanalization time	77.7 ± 32.07 (46–146)	58.6 ± 16.42 (38–82)	51.7 ± 24.53 (13–79)	0.14
Mean symptom‐to‐recanalization time	345 ± 165.38 (181–636)	272.67 ± 85.15 (193–413)	245.28 ± 78.25 (125–372)	0.26
EVT techniques				
Contact aspiration	5 (21.7%)	4 (17.4%)	4 (17.4%)	0.80
Combined technique (Aspiration + Stent retriever)	5 (21.7%)	2 (8.7%)	3 (13%)	
Recanalization outcomes				
mTICI 2c recanalization	0 (0%)	1 (4.3%)	0 (0%)	0.24
mTICI 3 recanalization	10 (43.5%)	5 (21.7%)	7 (30.4%)	0.24
Complication number	1^*^	1^**^	1^***^	
Clinical outcomes (mRS)				
3rd month mRS	3.1 ± 2.56	2.33 ± 2.25	3.57 ± 2.51	0.66
3rd month mRS 0	2 (8.7%)	1 (4.3%)	1 (4.3%)	0.95
3rd month mRS 0–1	5 (21.7%)	3 (13%)	1 (4.3%)	0.23
3rd month mRS 0–2	5 (21.7%)	4 (17.4%)	2 (8.7%)	0.37
3rd month mRS 0–3	5 (21.7%)	4 (17.4%)	3 (13%)	0.66
3rd month mRS 6	3 (13%)	1 (4.3%)	3 (13%)	0.58

Abbreviations: mRS, Modified Rankin Scale; mTICI, Modified Thrombolysis in Cerebral Infarction.

* During balloon angioplasty performed due to insufficient flow pattern at the proximal basilar artery after EVT, vertebral artery rupture, and hemorrhage occurred due to microwire manipulation.

** One case experienced a distal embolism.

*** In one case, hemorrhage occurred due to rupture of the left posterior cerebral artery (PCA) after stent retriever removal from the basilar artery via the left PCA.

**FIGURE 3:**
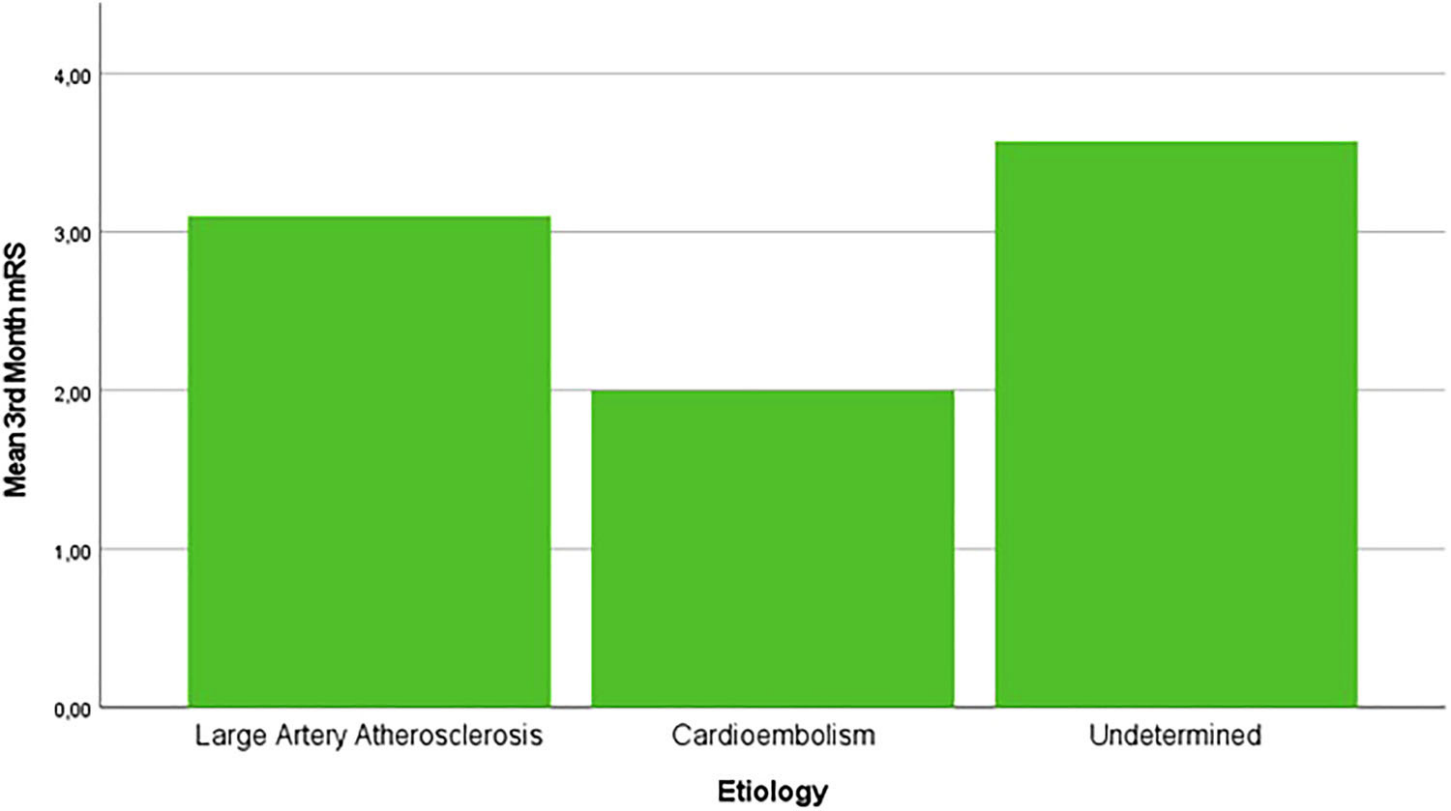
Mean 3rd month mRS values according to the etiology of the cases.

In terms of 3‐month functional outcomes, the cardioembolism group had the lowest mean mRS score (2.33 ± 2.25), although no statistically significant difference was observed among the etiological subgroups. The proportion of cases achieving 3rd month mRS 0 was 8.7% in the large artery atherosclerosis group, and 4.3% in both the cardioembolism and undetermined etiology groups. Regarding favorable outcomes (mRS 0–2) in 3rd month, these were observed in 21.7% (5 cases) of the large artery atherosclerosis group, 17.4% (4 cases) of the cardioembolism group, and 8.7% (2 cases) of the undetermined etiology group (Table 3, Figure 3). As for 3rd month mRS 6, it was noted in 13% of the large artery atherosclerosis group, 4.3% of the cardioembolism group, and 13% of the undetermined etiology group.

When the data were analyzed by the BAO site, the contact aspiration technique was used in 1 of the 5 cases with proximal occlusion, while the combined technique was used in the remaining 4 cases. In cases with distal occlusion, 52.2% (12 cases) underwent contact aspiration, and 26.1% (6 cases) underwent the combined technique. First‐pass successful recanalization rates, mean total intracranial passes, and complication rates according to occlusion site are shown in Table 4, with no significant differences between the groups.

**TABLE 4:** EVT techniques, number of intracranial passes, and procedural complication rates according to the localization of the occlusion in the basilar artery.

	Proximal basilar artery occlusion (*n* = 5)	Distal basilar artery occlusion (*n* = 18)	*p*
Contact aspiration	1 (4.3%)	12 (52.2%)	0.12
Combined technique (Aspiration + Stent retriever)	4 (17.4%)	6 (26.1%)	
First pass recanalization	3 (13%)	11 (47.8%)	0.96
Mean intracranial pass number	1.60 ± 0.89	1.55 ± 0.85	0.92
Complications	1^*^	2^**^	

* During balloon angioplasty performed due to insufficient flow pattern at the proximal basilar artery after EVT, vertebral artery rupture, and hemorrhage occurred due to microwire manipulation.

** In one case, hemorrhage occurred due to rupture of the left posterior cerebral artery (PCA) after stent retriever removal from the basilar artery via the left PCA.

When the cases were grouped according to the EVT technique, the first‐pass successful recanalization rate was 43.5% in the contact aspiration group and 17.4% in the combined technique group. The puncture‐to‐recanalization and symptom‐to‐recanalization times were longer in the combined technique group compared to the contact aspiration group, but these differences were not statistically significant (Table 5). The rate of good clinical outcomes was 26.1% in the contact aspiration group and 21.7% in the combined technique group.

**TABLE 5:** First pass recanalization rates, time metrics, and 3rd month mean mRS scores of the cases according to the techniques used in EVT.

	Contact Aspiration (*n* = 13)	Combined technique (Aspiration + Stent retriever) (*n* = 10)	*p*
First pass recanalization	10 (43.5%)	4 (17.4%)	0.1
Puncture‐to‐recanalization time (minutes)	57.15 ± 22.37 (13–96)	74.80 ± 32.55 (38–146)	0.13
Symptom‐to‐recanalization time (minutes)	289.30 ± 79.16 (181–436)	304.20 ± 178.87 (125–636)	0.81
mTICI 2c‐3 recanalization	13 (100%)	10 (100%)	
3rd month mRS (mean ± Sd)	3.38 ± 2.84	2.40 ± 1.64	0.31
3rd month mRS 0–1	5 (21.7%)	4 (17.4%)	0.94
3rd month mRS 0–2	6 (26.1%)	5 (21.7%)	0.85
3rd month mRS 0–3	6 (26.1%)	6 (26.1%)	0.68
3rd month mRS 6	6 (26.1%)	1 (4.3%)	0.08
Complications	2^*^ (15.4%)	1^**^ (10%)	

* A distal embolism in 1 case and during balloon angioplasty performed due to insufficient flow pattern at the proximal basilar artery after EVT, vertebral artery rupture and hemorrhage occurred due to microwire manipulation in 1 case.

** In one case, hemorrhage occurred due to rupture of the left posterior cerebral artery (PCA) after stent retriever removal from the basilar artery via the left PCA.

The mean mRS scores at 3 months were lower in the cardioembolic etiology and combined technique groups (Tables 3 and 5).

## 4 Discussion

BAO accounts for approximately 1% of all strokes, yet it is considered one of the most severe conditions due to its high mortality and disability rates, constituting around 10% of all large vessel occlusions (Yu and Higashida 2022). Prior to randomized controlled trials conducted after 2020, the importance and efficacy of EVT for BAO could not be clearly established. However, these trials have demonstrated the effectiveness of EVT in BAO. That being said, procedural risks associated with BAO and functional outcomes are emphasized to be worse in comparison to anterior system occlusions (Liu et al. 2020; Langezaal et al. 2021; Tao et al. 2022; Jovin et al. 2022; Nogueira et al. 2025).

In the 2022 BAOCHE randomized controlled trial, the average age of patients who underwent EVT for BAO was 65, and 73% of the patients were male (Jovin et al. 2022). In the ATTENTION trial, the average age of the EVT group was reported to be 66, with 66% of patients being male (Tao et al. 2022). The data in our study is consistent with the literature, with an average age of 60.86 and 73.9% male patients.

In the ATTENTION trial, patients with an NIHSS score ≥ 10 were included in the study (Tao et al. 2022), while in the BAOCHE trial, the inclusion criterion was NIHSS scores > 6 (Jovin et al. 2022). In contrast, in the BASICS trial, although the initial plan was to include patients with NIHSS > 10, the criteria were later broadened to include patients with NIHSS < 10 (Langezaal et al. 2021). In our study, NIHSS scores ranged from 2 to 38, with 87% of patients having a NIHSS score of ≥ 10 upon admission.

When examining the patient's medical history, HT emerged as the most common risk factor in our study. Similarly, the most frequently observed comorbidity in the literature is also HT (Liu et al. 2020; Tao et al. 2022; Jovin et al. 2022; Eren and Yücel 2021). When reviewing the etiological data of large studies on BAO, the BEST study found that 56% of patients had large artery atherosclerosis, 21% had cardioembolism, and 23% had undetermined/other causes (Liu et al. 2020). The ATTENTION study reported that 48% of patients had large artery atherosclerosis, 20% had cardioembolism, and 32% had undetermined/other causes (Tao et al. 2022). In our study, as in the literature, 43.5% of the cases had large artery atherosclerosis, 26.1% had cardioembolism, and 30.4% had undetermined causes.

Regarding the localization of BAO, a significant majority of cases in our study (78.3%) showed occlusion in the distal basilar artery. In contrast, large randomized controlled trials in the literature have reported a varying incidence of occlusion in the proximal basilar artery, with rates ranging between 30% and 50% (Liu et al. 2020; Langezaal et al. 2021). Recent studies have suggested that the recommended window for EVT in BAO is the first 24 h (Yu and Higashida 2022; Tao et al. 2022; Jovin et al. 2022). In our study, the symptom‐to‐recanalization times ranged from 125 to 636 min, with an average time of 295.78 ± 128.70 min. When comparing recanalization times by etiology, we observed significantly longer recanalization times in the large artery atherosclerosis group compared to the cardioembolism and undetermined etiology groups (Table 3). Although statistical significance could not be observed between groups due to the small number of cases, the numerical difference in the atherosclerotic group is noteworthy. The two cases with the longest puncture‐to‐recanalization times (146, 110 min) were both from the atherosclerotic group and underwent the combined technique. Large artery atherosclerosis‐related occlusions often present with a higher risk of intraprocedural reocclusion, necessitating additional interventional techniques. In addition, large artery atherosclerosis‐associated thrombi are usually rich in fibrin, which makes them more resistant to mechanical retrieval. Because of these reasons, recanalization time could be longer in this subgroup (Gory et al. 2018). These observations support the idea that EVT strategies tailored to the underlying stroke etiology—such as selecting thrombectomy techniques based on clot composition and/or etiology—may enhance procedural efficiency and improve outcomes in BAO. In the ATTENTION trial, the average puncture‐to‐recanalization time was reported as 1.2 h (Tao et al. 2022), while in the BAOCHE trial, this time was reported as an average of 85 min (Jovin et al. 2022). However, these studies did not provide data comparing recanalization times by etiology. Final successful recanalization (mTICI 2c‐3) was achieved in all cases in our study. When examining successful recanalization rates in the literature, the ATTENTION trial reported a rate of 93.3% (Tao et al. 2022), BAOCHE reported 88.1% (Jovin et al. 2022), BASICS reported 72% (Langezaal et al. 2021), and the BEST study reported 71% (Liu et al. 2020).

When reviewing thrombectomy techniques in the literature, the BEST study reported that 83% of patients underwent stent retriever use (Liu et al. 2020). In the BAOCHE trial, the distribution of thrombectomy techniques was as follows: 5% of patients received only the stent retriever, 34.8% received only aspiration, and 49.8% received the combined technique (aspiration + stent retriever) (Jovin et al. 2022). In our study, 43.5% of patients underwent the combined technique, while 56.5% underwent contact aspiration. In a 2018 study comparing contact aspiration and stent retriever techniques in BAO, it was found that contact aspiration, as the first technique, was associated with shorter procedure times and higher rates of complete reperfusion (Gory et al. 2018). Similar to the literature, we observed shorter procedure times in the contact aspiration group; however, the 3‐month average mRS scores in this group were higher than those in the combined technique group. Although, statistical differences between the groups were not observed. The poorer clinical outcome in the contact aspiration group, despite both groups having low and similar complication rates, suggests that this may not solely be attributable to complications.

In the ATTENTION study, 33% of patients had an mRS score between 0 and 2 at 3 months (Tao et al. 2022). The incidence of intracranial hemorrhage within 24–72 h was 14%, with a symptomatic hemorrhage rate of 5.3% (Tao et al. 2022). In the BAOCHE trial, 39% of patients had an mRS score between 0 and 2 at 3 months, with a symptomatic intracranial hemorrhage rate of 6%, and procedural complication rates (dissection, perforation, distal embolization) were reported to be 11% (Jovin et al. 2022). In a condition like BAO, which has a high mortality rate, a functional outcome of mRS 0–3 at 3 months is still considered clinically meaningful. In our study, the proportion of good functional outcomes (mRS 0–2) at 3 months was 47.8%, which is similar to the literature. The number of cases with excellent functional outcomes (mRS score 0–1) was 9 (34.1%). In these cases, the puncture‐to‐recanalization and symptom‐to‐recanalization times were 67.0 ± 23.48 min and 296.0 ± 137.74 min, respectively. By etiology, the 3‐month average mRS scores in the cardioembolism group were lower than in the large artery atherosclerosis and undefined etiology groups. Regarding complications, intracranial hemorrhage was observed in 3 cases (13%) within the first 24 h, with 2 of these being classified as symptomatic intracranial hemorrhage (8.7%). In addition, distal embolization was detected in only 1 case (4.3%), with no dissections, perforations, or other procedural complications observed. Two of these complications occurred in the contact aspiration group, while 1 occurred in the combined technique group (Table 5).

In conclusion, BAO constitutes the subgroup of large vessel occlusion with the highest mortality and morbidity rates. The data from randomized controlled trials emphasize the effectiveness of EVT in BAO, yet it is important to note the higher complication rates compared to anterior system occlusions. In our study, we found that the mean 3‐month mRS scores were lower in the cardioembolic etiology group. Moreover, an important finding that distinguishes our study from the literature is that although the combined technique group had longer recanalization times, they exhibited lower mean 3rd‐month mRS scores. As the number of randomized controlled trials and meta‐analyses increases, and as stent retrievers and aspiration catheters with advanced technologies and lower complication risks are developed, it is expected that mechanical thrombectomy in BAO will be recommended with higher levels of evidence in future guidelines.

Our study has several limitations. First, the small sample size limits the statistical power and generalizability of the results. Furthermore, the limited number of patients in each etiological subgroup made statistical comparisons between groups challenging. Larger, randomized controlled studies are needed to confirm and clarify the strength of our findings. Second, this was a single‐center, retrospective analysis, which may introduce selection and reporting biases.

## Author Contributions

**Halil Alper Eryilmaz**: conceptualization, data curation, project administration, resources, visualization. **Sena Boncuk Ulaş**: conceptualization, data curation, formal analysis, resources, writing – original draft, writing – review and editing, validation, software. **Bilgehan Atılgan Acar**: conceptualization, methodology, data curation, supervision, project administration, validation, resources, writing – review and editing.

## Ethics Statement

The study was conducted in accordance with the Declaration of Helsinki and approved by the Ethics Committee of Sakarya University Faculty of Medicine (Approval No: E‐43012747‐050.04‐438109‐06).

## Consent

Informed consent was obtained from all subjects involved in the study.

## Conflicts of Interest

The authors declare no conflicts of interest.

## Data Availability

Due to the nature of the research, due to ethical reasons supporting data is not available.
