# Correction to “Impact of Etiology, Thrombectomy Techniques, and Lesion Localization on Endovascular Treatment Success in Acute Basilar Artery Occlusion: A Single‐Center Retrospective Study”

**DOI:** 10.1002/brb3.70703

**Published:** 2025-08-13

Eryilmaz, H. A., S. Boncuk Ulaş, and B. A. Acar. 2025. “Impact of Etiology, Thrombectomy Techniques, and Lesion Localization on Endovascular Treatment Success in Acute Basilar Artery Occlusion: A Single‐Center Retrospective Study.” *Brain Behavior* 15: e70612. https://doi.org/10.1002/brb3.70612.

The funding statement for this article was missing. The below funding statement has been added to the article:

This study was supported by TUBITAK.

We apologize for this error.

